# Characterization of *Protosiphon botryoides* KNUA219 Isolated from Dokdo Island as a Potential Biofuel Resource

**DOI:** 10.4014/jmb.2411.11065

**Published:** 2025-02-25

**Authors:** Hae-Seo Noh, Jeong-Mi Do, Ho-Seong Suh, Su-Bin Park, Ho-Sung Yoon

**Affiliations:** 1 Department of Biology, Kyungpook National University, Daegu 41566, Republic of Korea; 2 School of Life Sciences, BK21 FOUR KNU Creative BioResearch Group, Kyungpook National University, Daegu 41566, Republic of Korea; 3 Integrated Blue Carbon Research Center, Advanced Bio-Resource Research Center, Kyungpook National University, Daegu 41566, Republic of Korea; 4 Biological Resources Research Department, Nakdonggang National Institute of Biological Resources (NNIBR), Sangju 37242, Republic of Korea

**Keywords:** Microalgae, *Protosiphon*, biomass, high-value products, biodiesel

## Abstract

The increasing demand for sustainable and eco-friendly energy sources has intensified research into alternative biofuel feedstocks. Microalgae, recognized for their rapid growth and production of high-value products, have emerged as promising candidates for third-generation biofuels. This study evaluates the potential of *Protosiphon botryoides* KNUA219, a microalga isolated from Dokdo Island, South Korea, as a biodiesel feedstock. Molecular and morphological analyses confirmed its identity, while growth experiments demonstrated its species-specific physiological characteristics, including an optimal pH range of 5−7, limited salinity tolerance, and high biomass productivity. Biochemical analysis revealed significant levels of carbohydrates (30.42 ± 1.65%), proteins (26.18 ± 1.14%), and lipids (14.86 ± 0.33%) in *P. botryoides* KNUA219, with glucose and galactose as the dominant monosaccharides. Fatty acid methyl ester profiling identified a lipid composition consisting of saturated (20.54%), monounsaturated (19.03%), and polyunsaturated fatty acids (42.65%), with palmitic, oleic, and linolenic acids as key components. Biodiesel quality assessments indicated compliance with critical standards for cetane number and cold filter plugging point, although optimization was required for iodine value and density. Proximate and ultimate analyses revealed favorable energy properties, including a high volatile matter content (88.94 ± 0.33%) and a calorific value of 23.11 ± 0.11 MJ/kg. These findings establish *P. botryoides* KNUA219 as a promising and sustainable resource for biodiesel production, while highlighting its potential for broader industrial applicability.

## Introduction

In 2023, biofuels accounted for 5.6% of global liquid fuel transport demand, with aviation and maritime sectors driving more than 75% of the new biofuel demand expected by 2030 [1]. This reflects a growing recognition of the urgent need to transition away from fossil fuels to mitigate climate change. The energy crisis and the increasing demand for environmentally friendly solutions are among the most pressing global challenges. Fossil fuel-based power generation is a significant contributor to greenhouse gas emissions and environmental degradation due to its reliance on high-carbon energy resources. In response, eco-friendly and renewable energy sources are gaining significant attention as viable alternatives [2]. This transition has led to the development and use of first-generation, second-generation, and third-generation biofuels. Biofuels offer notable advantages, including a lower carbon footprint, reduced byproduct generation compared to traditional fossil fuels, adaptability, and availability [3]. However, first-generation resources such as crop-based materials and second-generation resources such as lignocellulosic materials have limitations in areas such as competition with food resources, yield optimization, and pretreatment efficiency [4]. These challenges have led to the exploration of alternative resources for biofuel production, among which microalgae have emerged as a promising third-generation. Microalgae offer solutions to these limitations with their high lipid accumulation capacity, rapid growth rate, and adaptability to diverse environments [3, 5, 6]. Additionally, microalgae have ability to grow in non-arable land and utilize wastewater as a nutrient source further enhances cost efficiency and reduces competition with food resources, addressing significant economic challenges in biofuel production [7].

Microalgae are recognized as a source of high-value products, containing various bioactive compounds such as carotenoids, omega-3 and omega-6 fatty acids, amino acids, and proteins. These compounds are known for their antioxidant, anti-aging, and nutritional benefits, making microalgae a promising resource for nutraceutical and pharmaceutical applications [8]. However, the characteristics of these compounds can vary significantly across different microalgae species, highlighting the importance of species-specific research. The lipid accumulation ability of microalgae, which is crucial for the production of fatty acid methyl esters (FAMEs), directly impacts the quality of biodiesel. FAMEs are classified into saturated fatty acids (SFAs), monounsaturated fatty acids (MUFAs), and polyunsaturated fatty acids (PUFAs), each influencing the physical and chemical properties of biodiesel. Therefore, analyzing the characteristics of these bioactive compounds is essential for evaluating the potential of microalgae in biodiesel production and other industrial applications [9-11].

The microalga *P. botryoides* KNUA219 used in this study was isolated from soil collected on Dokdo Island, South Korea. This species is known to have the ability to survive in dry environments [12], and there have been reports of its survival in soil that had been dry for 43 years [13]. Therefore, this study aims to provide a comprehensive understanding of the characteristics of *P. botryoides* KNUA219 through a detailed analysis of its fundamental growth properties, responses to various stress environments, biochemical composition, and both proximate and ultimate analyses. By investigating these fundamental traits, this research seeks to evaluate the potential for biodiesel production and to provide foundational insights that may facilitate the optimization and enhancement of the viability of biodiesel production. This research will present the potential of *P. botryoides* KNUA219 as a new resource for biodiesel production.

## Materials and Methods

### Isolation and Identification

**Isolation and molecular identification.** The strain examined in this study was obtained from soil samples collected on Dokdo Island, East Sea, South Korea. To characterize the *P. botryoides* KNUA219 isolates, genomic DNA was extracted using a DNeasy Plant Mini kit (Qiagen, Germany), and molecular identification was performed using 18S rRNA, ITS, and *tufA* primers as markers. DNA sequences obtained in this study were compared using BLAST against the NCBI database. A phylogenetic tree was constructed using the MEGA X software package based on the BLAST results to elucidate the evolutionary relationships among these sequences.

**Morphological identification.** During the growth experiment, the morphology of the microalgae was observed on days 1, 3, 6, and 9 using a light microscope with a camera (Axiocam 208, Zeiss, Germany) at a magnification of 1,000×. For analysis of detailed surface morphology, samples were prepared by fixation using 100% glutaraldehyde and dehydration through a gradual increase in ethanol concentration. After platinum coating, the samples were observed using SEM (EM-30, Coxem, Republic of Korea).

### Growth Measurements

**Growth rate and biomass productivity.** The strain was inoculated into 500 ml of BG-11 medium in a 1L flask. The experiment was conducted at 25°C, 200 μmol/m^2^/s light intensity, 16:8 light/dark cycle, and 120 rpm (VS-202D shaker, Vision Scientific Co., LTD., Republic of Korea) with a mixture of 1−2.5% CO_2_ and filtered air injected. The growth rate was assessed daily by measuring the optical density (OD) at 680 nm with a spectrophotometer (Shimadzu UV-1900i, Shimadzu, Japan) and monitoring pH with a SevenDirect SD20 (Mettler Toledo, China). To determine biomass productivity, the culture solution (10 ml) at day 9 was vacuum-filtered using a pre-weighed 0.22 μm GF/C Whatman^TM^ filter paper (Thermo Fisher Scientific, UK) and dried at 105°C for 24 h in an oven (WiseVen WOF-W155, Daihan Scientific, Republic of Korea). Dry weight was determined by subtracting the weight of the pre-weighed filter from the total weight of the dried filter.

**Chlorophyll and total carotenoids.** Chlorophyll *a* and *b* and total carotenoids were measured at 3-day intervals. Aliquots of 2 ml culture were centrifuged (Labogene 1730R, Gyrozen, Republic of Korea) at 13,572 ×*g* for 3 min, and the pellets were suspended in 1 ml of 100% methanol (MeOH) and sonicated using a Branson Ultrasonics Sonifier 450 (Branson Inc., USA) at 40 kHz for 1 h (on-time 30 min). After 24 h incubation at 4°C in the dark, the absorbance of the supernatant was measured at 470, 653, and 666 nm to estimate pigment concentrations using the equations below [14]:

Chlorophyll *a* (Chl-*a*) = 15.65A_666_ – 7.34A_653_

Chlorophyll *b* (Chl-*b*) = 27.05A_653_ – 11.21A_666_

Total carotenoids = (1000A_470_ – 2.86Chl-*a*– 129.2Chl-*b*)/221

**Carotenoid analysis.** To extract the sample, 10 mg of biomass was treated with 1 ml of 60% KOH (w/v) and then washed with dichloromethane and MeOH [15]. The organic layer was filtered using a PVDF 0.22 μm syringe filter (PV1322, Chromdisc, Republic of Korea), concentrated using nitrogen gas, and dissolved in HPLC-grade MeOH for analysis using an Agilent 1200 series gradient HPLC system (Agilent Technologies, USA) with a photodiode array detector (200–800 nm). Solvents A (MeOH:tert-butyl methyl ether (TBME):water (81:15:4, v/v)) and B (7:90:3, v/v) were used as the mobile phase on a YMC carotenoid column (150 mm × 4.6 mm, 3 μm) at 35°C and a flow rate of 1 ml/min. This study employed lutein, zeaxanthin, β-carotene, fucoxanthin astaxanthin, and canthaxanthin, obtained from Sigma–Aldrich (USA), as the standard materials.

### pH and Salinity Stress Tolerance

pH and salt conditions were adjusted to evaluate the growth characteristics under stressful culture conditions. The initial pH conditions were set to 3, 5, 7, and 9. Salt tolerance experiments used 0 mM (control without NaCl), 10 mM, 50 mM, 100 mM, and 200 mM NaCl in BG-11 medium. Microalgae were inoculated into 100 ml of BG-11 medium in a 250 ml flask, ensuring that the initial OD (680 nm) was 0.1. The experiment was carried out for 12 days at 25°C, 200 μmol/m^2^/s of light intensity, 16:8 light/dark cycle, and 160 rpm. pH and OD (680 nm) were measured daily.

### Biochemical Composition

**Total carbohydrate and monosaccharide analysis.** After 9 days of growth experiments, the cell culture was harvested and freeze-dried (PVTFD20R, Ilshin, Republic of Korea) to obtain biomass powder. All subsequent experiments were conducted using this biomass.

Total carbohydrate content was analyzed by the phenol-sulfuric acid method [16]. Briefly, 50 mg of biomass was acid-hydrolyzed with 2.5 ml of 2 N sulfuric acid in a boiling water bath (LWB-122D, Daihan Labtech, Republic of Korea) for 3 h, neutralized with sodium carbonate, diluted to 1 mg/ml, and centrifuged. The supernatant was filtered using a 0.2 μm syringe filter (Minisart NML, Sartorius, Germany) and then incubated with 50 μl of 80%phenol solution (w/v) and 5 ml of sulfuric acid. OD was measured at 490 nm.

For monosaccharide analysis, HPLC was performed with the filtered sample from the above process using an Alliance HPLC system (Waters Co., USA) with a refractive index (RI) detector on a Sugar-Pak column (300 mm × 6.5 mm, 10 μm). A 20 μl sample was injected into the mobile phase of 0.01 M Ca-EDTA (50 mg/l in dH_2_O) at a flow rate of 0.5 ml/min and 90°C. Sucrose, maltose, lactose, glucose, xylose, galactose, mannose, fructose, fucose, arabinose, and mannitol were employed as the standards and were obtained from Sigma–Aldrich.

**Total protein and free amino acid analysis.** Total protein content was quantified using a Pierce^TM^ BCA Protein Assay Kit (Thermo Fisher Scientific) after sample pretreatment. A 20 mg biomass sample was sonicated for 1 h (5-sec intervals) with 1 ml of RIPA Lysis Buffer (1X) with EDTA (GenDEPOT, USA) and incubated at 100°C for 30 min. The supernatant was mixed with 10% (w/v) trichloroacetic acid (TCA) in ice-cold acetone. The pellet was then washed and dissolved in 2 ml each of 8 mol/l urea (pH 8.8) in Tris-HCl 60 mmol/l and BCA working reagent. Absorbance at 562 nm was measured [17]. Free amino acid analysis was also conducted. For acid hydrolysis, 0.1 g of biomass was mixed with 1 ml each of 0.1 N HCl and 5% TCA. The supernatant was filtered, and the absorbance was measured at 440 and 570 nm wavelengths using an Amino Acid Autoanalyzer (LA8080, Hitachi, Republic of Korea).

**Total lipid and fatty acid methyl ester analysis.** Total lipid content was analyzed using the sulfo-phospho-vanillin method [18]. A biomass sample of 10 mg suspended in 1 ml of distilled water was boiled at 100°C for 10 min with 2 ml of concentrated (98%) sulfuric acid. After cooling on ice, 5 ml of phosphor-vanillin reagent was added. The absorbance was measured at 530 nm.

FAME content was determined using Breuer's method [19]. Briefly, 10 mg of biomass was disrupted using a Vortex-Genie (Scientific Industries, USA) and a sonicator. The extracted lipids were transesterified using 3 ml of MeOH containing 5% (v/v) sulfuric acid, and FAMEs were extracted with hexane. After filtration through a PVDF 0.22 μm syringe filter, the composition was analyzed using a GC/MS (Agilent 7890B-5977B, USA) with a DB-FFAP column (30 m × 250 μm × 0.25 μm) and the electron ionization method.

### Biodiesel Quality Assessment

Biodiesel quality was assessed by calculating various fuel properties, including the saponification value (SV), iodine value (IV), cetane number (CN), degree of unsaturation (DU), long-chain saturated factor (LCSF), cold filter plugging point (CFPP), oxidative stability (OS), kinematic viscosity (υ), and density (ρ). These calculations were derived from the FAME profile of the biodiesel using the equations provided below [20-22]:

SV = Σ (560 × P)/MW

IV = Σ (254 × P × D)/MW

CN = (46.3 + 5458/SV) – (0.225 × IV)

DU = Σ MUFA + (2 × PUFA)

LCSF = (0.1 × C16) + (0.5 × C18) + (1.0 × C20) + (1.5 × C22) + (2.0 × C24)

CFPP = (3.1417 × LCSF) – 16.477

OS = 117.9295/X + 2.5905 (0<100)

ln(υ) = Σ -12.503 + 2.493 × ln(MW) – 0.178 × D

ρ = Σ 0.8463 + 4.9/MW + 0.0118 × D

In the equations, P is the percentage of each fatty acid content; MW is the molecular weight; D is the number of double bonds; C16, C18, C20, C22, and C24 represent the percentage content of C16:0, C18:0, C20:0, C22:0, and C24:0, respectively; X is the linoleic and linolenic acid content in each FAME value.

### Proximate Analysis and Ultimate Analysis

Proximate analysis of 4 mg of biomass was performed using a thermal analyzer (DTG-60A, Shimadzu, Japan). The sample was heated from 50°C to 900°C at a rate of 10°C/min in a platinum pan, with 30 mg of α-alumina (α-Al_2_O_3_) powder as a reference material. The moisture, volatile matter, and ash contents were determined following previous study [23]. Moisture content was calculated by measuring the mass reduction of biomass after drying at 105°C. Volatile matter was assessed by the mass loss after drying, and ash content was determined from the residual mass after combustion reactions. Ultimate analysis of biomass was conducted using an elemental analyzer (FlashSMART, Thermo Fisher Scientific, Italy) to determine the carbon, hydrogen, oxygen, nitrogen, and sulfur content. Based on the proximate and ultimate analyses, the calorific value (CV) was calculated using the equation provided below [24]:

CV (MJ/kg) = 0.3278C + 1.419H + 0.09257S – 0.1379O + 0.637

### Statistical Analysis

All experiments were performed in triplicate, and the average value of each set of replicates was used as the result. The results were presented as the mean ± standard deviation, with error bars visually representing the variability.

## Results and Discussion

### Identification of KNUA219

**Fig. 1:**
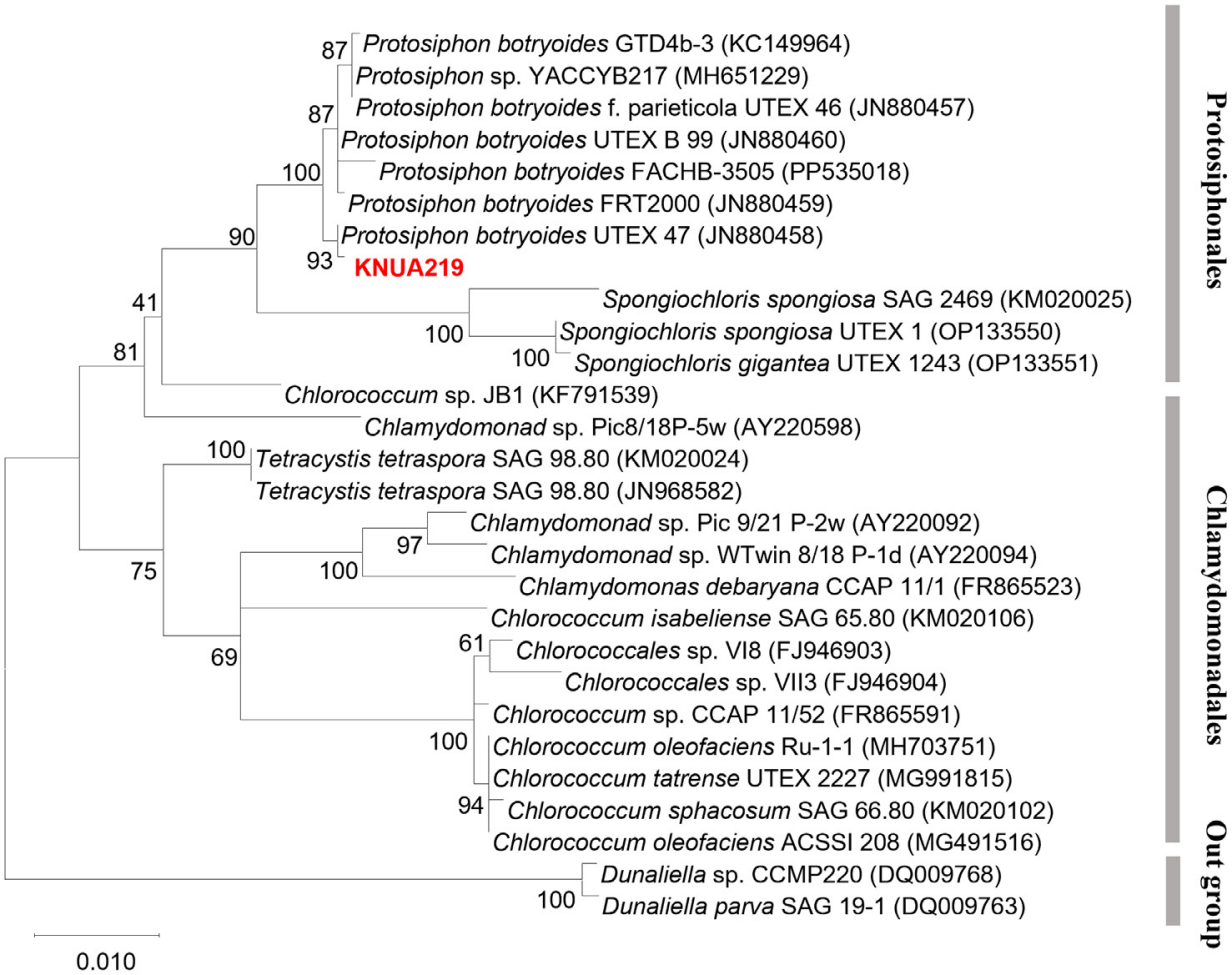
Phylogenetic relationship of *P. botryoides* KNUA219 and its closely related species inferred from the 18S rRNA sequence data. The Maximum Likelihood method with Kimura 2-parameter model was used, and bootstrap analysis was performed with 1000 replicates. The scale bar indicates a 1% difference in nucleotide sequences.

**Molecular identification.** NCBI similarity analysis using 18S rRNA, ITS, and *tufA* primers indicated that KNUA219 was similar to *P. botryoides* (Table S1). Phylogenetic analysis was performed using the maximum likelihood method. In the phylogenetic tree (Fig. 1), two major clades were formed, the Protosiphonales clade and the Chlamydomonadales clade, with *Dunaliella* species as the outgroup. KNUA219 was clustered with *Protosiphonales* species and had the closest phylogenetic relationship to *P. botryoides* UTEX 47. KNUA219 was identified as *P. botryoides*.

**Fig. 2:**
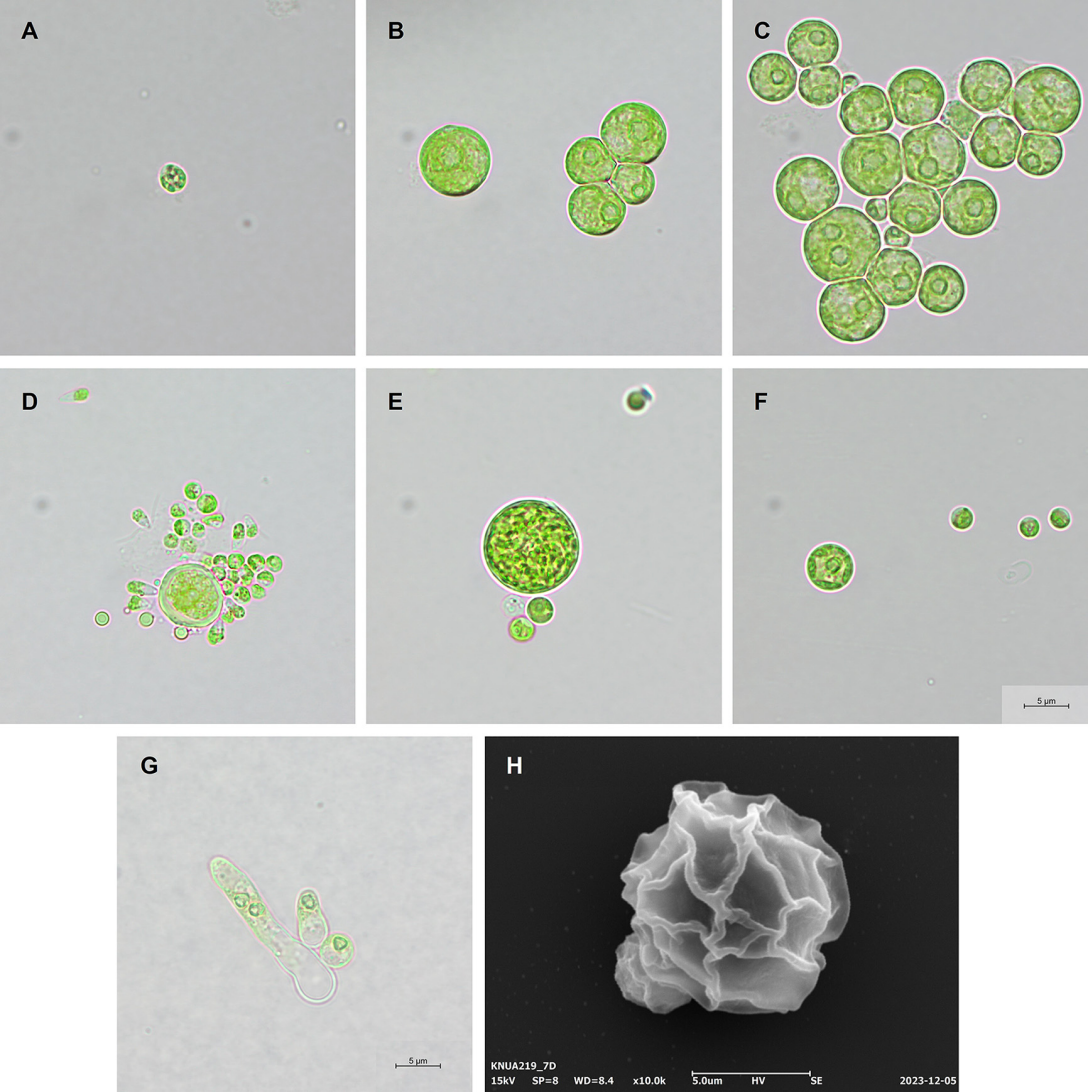
Microscopic images of *P. botryoides* KNUA219. Light microscope images captured at 1000× magnification from liquid medium culture: (**A**) Day 1; (**B, C**) Day 3; (**D**) Day 6; (**E, F**) Day 9. (**G**) Light microscope image captured at 1000× magnification from agar medium culture. (**H**) Scanning electron microscope (SEM) image. All experiments were conducted at 25°C with 200 μmol/m²/s light intensity and 16:8 light/dark cycle. Liquid cultures used 100 ml of BG-11 medium at 160 rpm, while agar cultures were prepared with BG-11 agar.

**Morphological identification.** In liquid medium, *P. botryoides* KNUA219 exhibited a spherical growth pattern from the first day, with an increase in size over time (Fig. 2A-2F). By the sixth day, motile biflagellate zoospores were observed (Fig. 2D). This spherical morphology is indicative of the typical growth pattern in liquid media, with the coenocytic form illustrated in Fig. 2E. Additionally, Fig. 2H depicts the surface characteristics of the spherical cells in liquid medium. Conversely, in solid medium, the development of root-like structures (rhizoid) and tubular forms, which are less visible in liquid medium, was prominently observed (Fig. 2G). This observation suggests that rhizoid development is enhanced in solid media, reflecting an adaptation to the physical conditions of the soil [25].

*P. botryoides* occurs in clusters on the soil surface and maintains unicellular and coenocytic forms in liquid media [12, 26]. In solid media, it develops tubular and rhizoidal structures to optimize survival, reflecting adaptations to environmental factors such as thigmotropism and phototropism [25].

### Growth Measurements

**Fig. 3:**
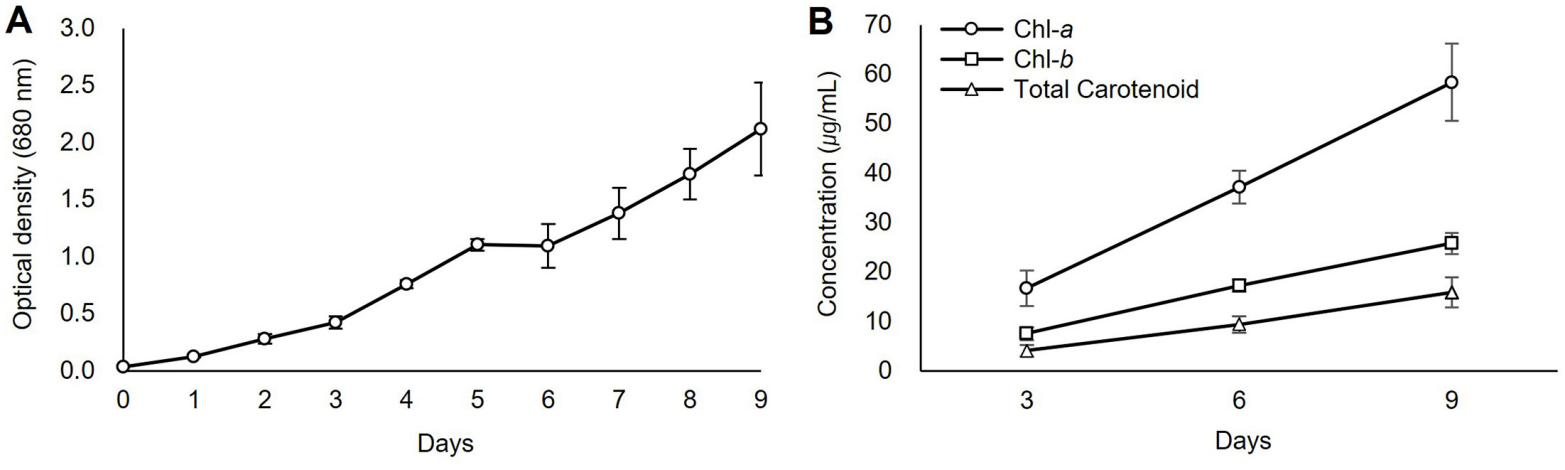
Growth and pigment variation in *P. botryoides* KNUA219. (**A**) Growth curve and (**B**) changes in pigment concentrations (chlorophyll *a* (Chl-*a*), chlorophyll *b* (Chl-*b*), total carotenoids) during 9 days of cultivation in 500 ml of BG-11 medium at 25°C, 200 μmol/m^2^/s light intensity, 16:8 light/dark cycle, 120 rpm, and 1−2.5% CO_2_ injection.

The initial OD of the microalgae culture was 0.035 ± 0.003. During 9 days of cultivation, the OD steadily increased, reaching a final value of 2.120 ± 0.408 (Fig. 3A). The dry weight was measured on the 9^th^ day, which was the last day, and the biomass productivity was found to be 1.98 ± 0.03 g/l. The 9-day experimental period was determined to capture the exponential growth phase, which represents the most active period of microalgae. This time point was selected based on previous studies of *P. botryoides*, which indicated a decline in growth rate after day 8 [27].

Over three days, the chlorophyll *a* level increased from 16.73 μg/ml to 58.39 μg/ml, the chlorophyll *b* level increased from 7.59 μg/ml to 25.80 μg/ml, and total carotenoids increased from 4.13 μg/ml to 15.91 μg/ml (Fig. 3B). This indicates that the photosynthetic ability of the microalgae improved as it grew. Analysis of each carotenoid component confirmed that the lutein content was 2.459 mg/g and the β-carotenoid content was 0.404 mg/g.

### pH and Salinity Stress Tolerance

**Fig. 4:**
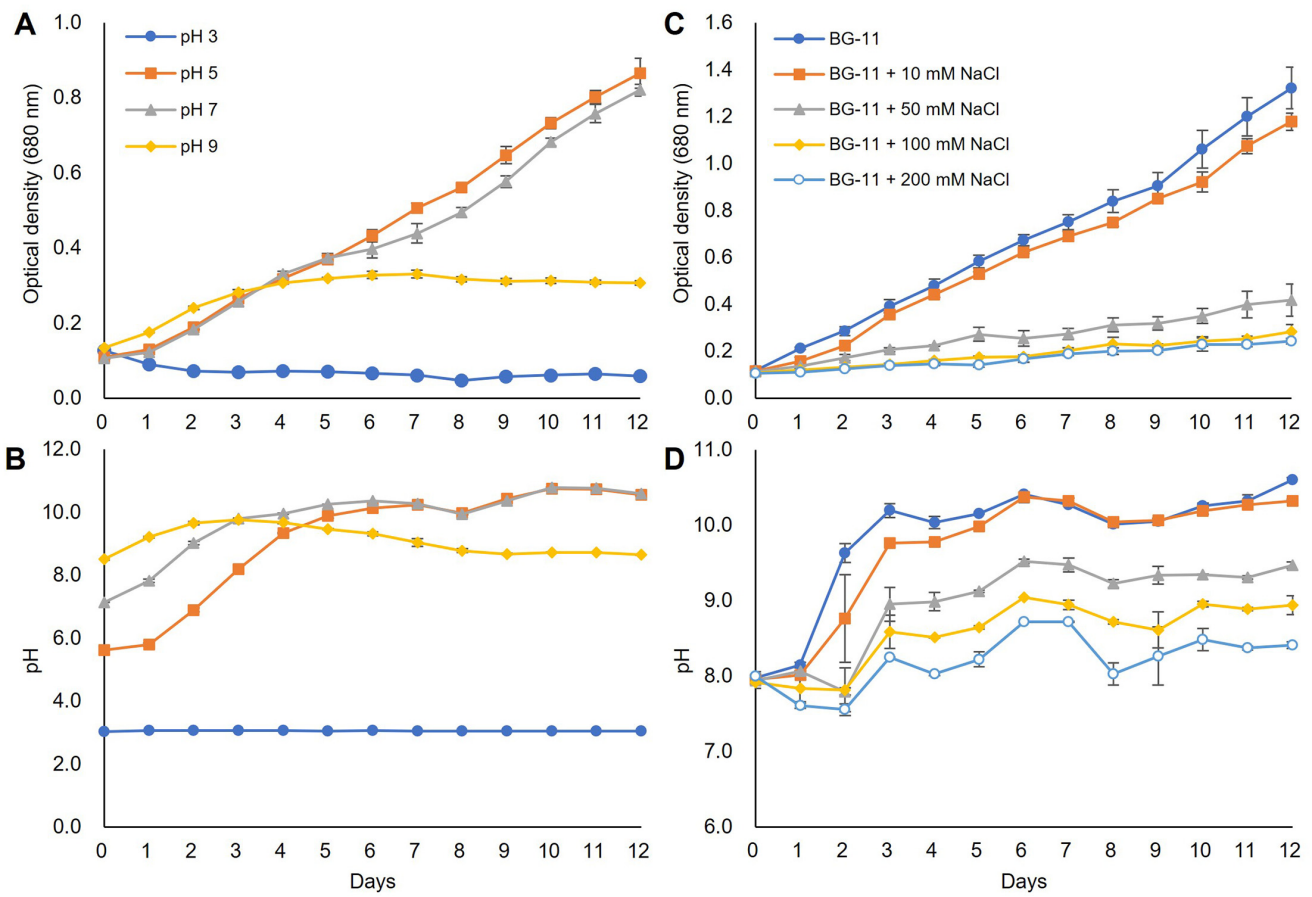
Growth parameters of *P. botryoides* KNUA219. (**A**) Optical density and (**B**) pH under different initial pH conditions; (**C**) Optical density and (**D**) pH under varying salinity conditions. All experiments were conducted at 25°C, 200 μmol/m^2^/s light intensity, 16:8 light/dark cycle, and 160 rpm.

This study investigated the stress tolerance of *P. botryoides* KNUA219 by examining its growth response to varying pH (Fig. 4A and 4B) and salinity (Fig. 4C and 4D) conditions for 12 days. In pH 3 medium, the microalgae died immediately, with no observed changes in OD and pH. At pH 5, the microalgae exhibited normal growth, similar to the control (pH 7) BG-11 medium. While initial growth, indicated by an increase in OD of approximately 0.2, was observed at pH 9, growth ceased after 4–5 days. This growth inhibition is likely attributable to the predominance of carbonate (CO_3_^2-^) under alkaline conditions, which represents a less readily available carbon source for most microalgae [28]. Furthermore, elevated concentrations of carbonate (CO_3_^2-^) may exert toxic effects, induce protein denaturation, and inhibit nutrient uptake, thereby disrupting cellular metabolic processes [29]. Interestingly, the pH in both pH 5 and pH 7 media increased despite microalgal growth, unlike the pH 9 medium. This suggests that the pH increase observed in pH 5 and pH 7 media resulted from a substance produced by the growing microalgae, rather than from the addition of NaOH. Specifically, this increase occurs due to the absorption of carbon dioxide (CO_2_) and the subsequent formation of carbonic acid (H_2_CO_3_), which leads to the consumption of hydrogen ions (H^+^) and the accumulation of bicarbonate ions (HCO_3_^-^) during the photosynthesis process of algae [30]. The results of this study show that *P. botryoides* KNUA219 has a high growth rate in the pH range of 5 to 7. Although previous studies have indicated pH 6–7 as the optimal pH for *P. botryoides* [25], this study demonstrates its capacity for substantial growth even under slightly acidic conditions at pH 5, suggesting a degree of tolerance to acidic environments.

BG-11 medium was supplemented with varying concentrations of NaCl (10 mM, 50 mM, 100 mM, and 200 mM) to evaluate the salinity tolerance of the microalgae. Growth in 10 mM NaCl medium was comparable to that in the control BG-11 medium, indicating effective growth under relatively low salinity conditions. However, the microalgae exhibited minimal growth in media containing 50 mM to 200 mM NaCl. Previous studies reported that germination and growth typically decline at salinity levels above 1% and are almost completely inhibited at 2%or higher [12]. This study demonstrates that *P. botryoides* KNUA219 can maintain substantial growth at 0.58%salinity (10 mM NaCl), underscoring its ability to adapt to slightly elevated salinity levels. Further investigation using intermediate NaCl concentrations (*e.g.*, 20 mM, 30 mM, and 40 mM) would be beneficial for accurately determining its upper salinity tolerance and comprehensively characterizing its growth response across a broader salinity range.

Microalgae exhibit significant variation in growth rate and productivity depending on various environmental factors such as salinity, nutrients, and temperature. Generally, microalgae prefer a pH of around 6 to 8 [31] and can grow at varying salinity levels depending on the species. For example, *Dunaliella salina* is found in high-salt environments and shows optimal growth under 2 M NaCl conditions [32]. However, cultivating microalgae in stressful environments for increased lipid production is actively being utilized as a strategy for biofuel production [33, 34]. Excessive stress, nonetheless, can decrease biomass production. Therefore, it is important to establish the optimal stress level to maximize lipid production without reducing biomass.

### Biochemical Composition

Biochemical composition analysis was conducted to identify the fundamental characteristics of *P. botryoides* KNUA219. Carbohydrates were the most abundant at 30.42 ± 1.65%, followed by proteins (26.18 ± 1.14%), and lipids (14.86 ± 0.33%). To determine which carbohydrates were most prevalent, an analysis of monosaccharides was performed. The results showed that glucose was the most abundant monosaccharide, with a concentration of 2,072 μg/ml, followed by galactose at 414 μg/ml. No other monosaccharides were reliably detected beyond the detection limit, suggesting either their absence or their presence in trace amounts. Glucose, a product of photosynthesis, plays a critical role as an energy reserve within the metabolic processes of microalgae. Although studies on the cell wall composition of *P. botryoides* are limited, the presence of galactose in the cell walls of other microalgae, such as *Chlamydomonas* [35], suggests a plausible structural role for galactose in this species. Therefore, the detection of glucose and galactose can be reasonably interpreted as indicative of their roles in metabolism and as structural components.

Amino acid analysis was conducted to characterize the protein profile (Table 1). Amino acids are important in physiological metabolism. Essential amino acids are amino acids that cannot be synthesized by the human body and must be obtained through dietary intake. The analysis revealed the presence of valine, leucine, lysine, isoleucine, histidine, and tyrosine, suggesting the potential application of this strain as feedstock in food and in the pharmaceutical industry. However, non-essential amino acids are also important as they are involved in biological reactions such as protein synthesis and energy production. Among them, glutamic acid, the most abundant amino acid, can be used as a natural flavor enhancer and is valued in the pharmaceutical industry for its role as a neurotransmitter in brain function [36]. Proline, identified as the most abundant amino acid, is classified as a conditionally essential amino acid that must be consumed under specific conditions. In plants, proline contributes to osmotic regulation and alleviation of oxidative stress. The results of this experiment suggest that CO_2_ injection enhanced the growth and metabolic activity of microalgae, thereby promoting proline accumulation [37]. The presence of essential, non-essential, and conditionally essential amino acids underscores the potential value of this strain across various industries.

The lipid and fatty acid profiles are crucial indicators of the potential and efficiency of microalgae as biofuels. This is because FAMEs, produced through the transesterification of fatty acids, serve as biodiesel [10]. The composition of FAMEs, including fatty acids with carbon chain lengths ranging from 14 to 18, is presented in Table 2. SFAs account for approximately 20.54% of the fatty acid content, with palmitic acid (C16:0) being the most abundant. MUFAs are mainly composed of oleic acid (C18:1) and account for 19.03% of the total. PUFAs have the highest proportion at 42.65%, with linolenic acid (C18:3, ω3) and C16:4 as the main components. In the case of *P. botryoides* KNUA219, palmitic (C16:0), stearic (C18:0), oleic (C18:1), linoleic (C18:2), and linolenic acids (C18:3, ω3), typically found in biodiesel [38], account for 60.873% of the total FAMEs.

FAME demonstrates significant potential across various industrial and commercial applications. Palmitic acid (C16:0) is utilized in cosmetics, and oleic acid (C18:1) serves as a solubilizer in aerosols, food supplements, drug excipients, and emulsifiers, playing a diverse role in the pharmaceutical and food industries. Linolenic acid (C18:3, ω3) contributes to cell membrane synthesis and tissue regeneration and is employed in the cosmetic and therapeutic sectors for skin revitalization [39, 40].

### Biodiesel Quality Assessment

FAMEs hold considerable promise not only for potential applications in soaps, emulsifiers, beauty products, and healthcare but also for biodiesel production. The quality of biodiesel is assessed through various parameters including SV, IV, CN, CFPP, oxidation stability, viscosity, and density. These parameters are determined by the FAME profile and are influenced by molecular structure, chain length, saturation, unsaturation, and the positions of the double bonds [41]. *P. botryoides* KNUA219 CFPP, oxidation stability, and viscosity values met the European EN14214 and American ASTM D6751 standards (Table 3). However, IV and density did not meet the European standards. The CN, an important factor indicating the ignition performance of diesel engine fuel, fell slightly short of the European standard but was higher than the American standard of 45. The reduction in CN is influenced by elevated levels of PUFAs and the resulting high IV. Fatty acids exhibit an antagonistic relationship in biodiesel properties, enhancing specific attributes while simultaneously degrading others. For instance, a low IV improves combustibility and efficiency but can adversely affect low-temperature flow characteristics [11]. In contrast, a high degree of unsaturation, characterized by low melting points, enhances the cold flow properties of biodiesel, thereby improving its performance in cold climates [9, 42]. This unsaturation also provides advantages in biolubricant applications, where a low viscosity and high fluidity are essential [43, 44]. Adjusting the fatty acid profiles associated with high IV could further optimize the lubricity and viscosity index of biodiesel, extending its applicability to specialized lubricant markets beyond traditional uses.

### Proximate Analysis and Ultimate Analysis

Proximate and ultimate analyses revealed the elemental composition of the biomass, providing insights into its suitability and quality as a biofuel feedstock (Table 4). Proximate analysis determined the moisture, volatile matter (VM), and ash values, while the calorific value (CV) was calculated based on the elemental composition obtained from the ultimate analysis. The moisture content was found to be 5.42 ± 0.79%, which is below the acceptable level of 10%. If the moisture content exceeds 10%, the biomass quality is considered to be low as it interferes with the conversion process, and an additional costly drying stage is required [23, 45]. *P. botryoides* KNUA219 exhibits a lower ash content compared to the average microalgae biomass ash content of 6−15% [45]. While previous generations of biofuel feedstocks typically exhibit VM contents of up to 80% (crop residue: 63−80%; wood: 72−78%) [46], *P. botryoides* KNUA219 has a higher VM content of 88.94 ± 0.33%. The high VM content and carbon content contribute to a CV of 23.11 ± 0.11 MJ/kg, which is higher than the 14.4−17.5 MJ/kg CV of common crop-based biofuel feedstocks such as maize, wheat, and sunflower [47]. High VM content increases the fuel reactivity and lowers ignition temperature, thus enhancing its potential as a liquid biofuel. This fuel is more reactive, facilitating combustion, and can also have a higher CV. Conversely, a high ash content can slow down decomposition and energy conversion rates, potentially leading to a lower CV [45]. The CV of biofuels is primarily influenced by carbon and hydrogen content, with higher values indicating higher energy content [48, 49].

The low ash content, high VM, and high CV of *P. botryoides* KNUA219 suggest its suitability for bioenergy production.

## Supplemental Materials

Supplementary data for this paper are available on-line only at http://jmb.or.kr.



## Figures and Tables

**Table 1:** The concentrations (μg/ml) of free amino acids.

Amino acid	Component	Concentration (μg/ml)
Essential	Valine (Val)	127.334
	Leucine (Leu)	112.864
	Lysine (Lys)	102.610
	Isoleucine (Ile)	73.923
	Histidine (His)	33.478
	Tyrosine (Tyr)	56.700
Conditionally essential	Proline (Pro)	2660.275
	Arginine (Arg)	259.471
	Glycine (Gly)	90.705
Non-essential	Glutamic Acid (Glu)	2131.245
	Alanine (Ala)	1319.938
	g-ABA	489.140
	EOHNH_2_	176.066
	Aspartic acid (Asp)	147.020
	Ornithine (Orn)	132.483
	Citrulline (Cit)	57.098
	NH_3_	16.271

**Table 2:** Fatty acid methyl esters (FAMEs) composition of *P. botryoides* KNUA219 (wt% of total FAMEs).

	*P. botryoides* KNUA219 (%)
Total lipid	14.86 ± 0.33
C14:0	0.284
C15:0	0.075
C16:0	19.067
C16:1	1.727
C16:2	3.472
C16:3	1.236
C16:4	11.398
C18:0	1.112
C18:1	17.306
C18:2	7.900
C18:3 (ω3)	15.489
C18:3 (ω6)	1.418
C18:4	1.735
SFAs	20.54
MUFAs	19.03
PUFAs	42.65

The culture was cultivated in BG-11 medium for 9 days at 25°C, 200 μmol/m²/s light intensity, a 16:8 light/dark cycle, and 120 rpm, with a CO_2_ injection of 1−2.5%. The freeze-dried biomass obtained from this culture was used for analysis.

**Table 3:** Biodiesel quality properties of *P. botryoides* KNUA219, calculated by FAMEs composition, compared with EN14214 and ASTM D6751 standards.

	*P. botryoides* KNUA219	EN14214	ASTM D6751
Saponification value (mg KOH / g)	163.65		
Iodine value (g I_2_ / 100 g fat)	134.51	≤ 120	
Cetane Number	49.39	≥ 51	≥ 45
Degree of unsaturation	104.3		
Cold filter plugging point (°C)	-8.7	-20 − 0	
Oxidation stability (110°C, h)	7.3	≥ 6	≥ 3
Kinematic viscosity (mm^2^ / s)	3.67	3.5	1.9 − 6.0
Density (15°C) (g / cm^3^)	0.89	0.872 − 0.878	

**Table 4:** Proximate and ultimate analyses of *P. Botryoides* KNUA219 biomass.

	*P. Botryoides* KNUA219
Proximate analysis (wt%)	
Moisture	5.42 ± 0.79
Volatile matter	88.94 ± 0.33
Ash	5.64 ± 1.11
Ultimate analysis (wt%)	
Carbon (C)	50.72 ± 0.03
Hydrogen (H)	7.06 ± 0.06
Oxygen (O)	30.47 ± 0.10
Nitrogen (N)	5.79 ± 0.01
Sulfur (S)	0.31 ± 0.02
CV * (MJ/kg)	23.11 ± 0.11

* Calorific Value
