# Supplementary material for: Characterization of Protosiphon botryoides KNUA219 Isolated from Dokdo Island as a Potential Biofuel Resource
Source: J Microbiol Biotechnol. 2025 Feb 25;35:e2411065. doi: 10.4014/jmb.2411.11065 (PMC11896802; doi:10.4014/jmb.2411.11065)
Supplement: Supplementary file 1 [file jmb-35-e2411065-supple.pdf]

J. Microbiol. Biotechnol. 2025. 35: e2411065

https://doi.org/10.4014/jmb.2411.11065

Supplementary Table

Table S1. Results of BLAST searches using 18S rRNA, ITS and *tuf*A gene sequences of strain KNUA219.

Marker gene	Length (bp)	Closest match (GenBank accession No.)	Query cover (%)	Identification (%)
18S rRNA	1763	*Protosiphon botryoides* FRT2000 (JN880459)	99	99.60
ITS	540	*Protosiphon botryoides* UTEX99 (LC095629)	68	94.14
*tuf*A	926	*Protosiphon botryoides* (MG778499)	99	97.72
